# Efficacy and safety of traditional Chinese herbal formula combined with western medicine for gastroesophageal reflux disease: A protocol for systematic review and meta-analysis

**DOI:** 10.1097/MD.0000000000022454

**Published:** 2020-10-09

**Authors:** Wuhong Lin, Guihua Huang, Xirong Liu, Huasheng Lin, Heng Zhou, Chunbing Feng, Tingshuai Wang, Renjiu Liang

**Affiliations:** a The School of Chinese Medicine, Hunan University of Chinese Medicine, Changsha, Hunan Province; b Department of Spleen, Stomach, and Liver Disease, The First Affiliated Hospital of Guangxi University of Chinese Medicine; c Emergency Department of Yulin Hospital of Traditional Chinese Medicine; d Graduate School, Guangxi University of Chinese Medicine, China.

**Keywords:** Chinese herbal formula, gastroesophageal reflux disease, meta-analysis, protocol, systematic review

## Abstract

**Background:**

The combined therapy of Chinese herbal formula and western medicine against gastroesophageal reflux disease (GERD) could significantly improve the clinical effect, reduce the recurrence rate and the side effects of western medicine, and even reduce the dosage and course of treatment of western medicine. This study tried to systematically evaluate the efficacy and safety traditional Chinese herbal formula combined with western medicine in the treatment of GERD.

**Methods:**

Randomized controlled trials of traditional Chinese herbal formula combined with western medicine for GERD patients will be systematically searched using the PubMed, Embase, Medline, Cochrane Library, China National Knowledge Infrastructure (CNKI), Wanfang database, Chongqing VIP Chinese Science and Technology Periodical Database, and Chinese Biological and Medical database (CMB) until Aug. 28, 2020. Two researchers will perform data extraction and risk of bias assessment independently. Statistical analysis will be conducted in RevMan 5.3.

**Results:**

This study will summarize the present evidence by exploring the efficacy and safety of traditional Chinese herbal formula combined with western medicine in the treatment of GERD.

**Conclusions:**

The findings of the study will help to determine potential benefits of traditional Chinese herbal formula combined with western medicine against GERD.

**Ethics and dissemination:**

The private information from individuals will not be published. This systematic review also will not involve endangering participant rights. Ethical approval is not required. The results may be published in a peer-reviewed journal or disseminated in relevant conferences.

**OSF Registration number:**

DOI 10.17605/OSF.IO/RSAVF.

## 1 Introduction

Gastroesophageal reflux disease (GERD), also known as acid reflux, is a condition in which stomach contents rise up into the esophagus, resulting in typical symptoms of the taste of acid in the back of the mouth, heartburn, chest pain, and dysphagia.^[1,2]^ At present, Barrett esophagus, nonerosive reflux disease, reflux esophagitis are the main clinical types. The prevalence varies in different areas, and it increases with age.^[3–5]^ It reduces patients’ quality of life, and it is associated with a strong and severity-dependent increased risk of esophageal adenocarcinoma.^[6,7]^

The treatment options include lifestyle changes, medications, and sometimes surgery. The primary medications used for GERD include proton-pump inhibitors, H2 receptor blockers, prokinetics, and antacids, in which acid suppression therapy is a common response to GERD symptoms. However, long-term use of the medications could cause acute and chronic kidney damage, magnesemia, and fractures. Also, acid suppression therapy alone can only improve symptoms such as acid reflux and heartburn, but cannot improve gastric motility and cure it. For thousands years, with the advantages of fewer adverse reactions and individualized treatment, traditional Chinese medicine has been widely used against GERD in China.^[8]^ Clinical studies have proved the efficacy and safety of Chinese herbal formula in treating GERD, and they appeared to be maintained for a longer period of time.^[9,10]^

In recent years, it has been discovered that the combined therapy of Chinese herbal formula and western medicine against GERD has great advantages. It could significantly improve the treatment effect, reduce the recurrence rate and the side effects of western medicine, and even reduce the dosage and course of treatment of western medicine. In order to explore the efficacy of the combined therapy in the treatment of GERD, this study tried to systematically evaluate the efficacy and safety traditional Chinese herbal formula combined with western medicine in the treatment of GERD.

## 2 Methods

### 2.1 Study registration

This protocol of systematic review and meta-analysis has been drafted under the guidance of the preferred reporting items for systematic reviews and meta-analyses protocols (PRISMA-P).^[11]^ Moreover, it has been registered on open science framework (OSF) on August 28, 2020 (Registration number: DOI 10.17605/OSF.IO/RSAVF).

### 2.2 Ethics

Ethical approval is not required because of no patient recruitment and personal information collection, and the data included in the study are derived from previous published literature.

### 2.3 Inclusion criteria for study selection

#### 2.3.1 Type of studies

Randomized controlled trials (RCTs) of Chinese herbal formula combined with western medicine on the treatment of GERD will be included, with the language limited to Chinese and English.

#### 2.3.2 Type of participants

All the included cases conform to the diagnosis of GERD (diagnosed with any recognized diagnostic criteria in the primary literature), regardless of nationality, race, age, gender, and source of cases.

#### 2.3.3 Type of interventions

The study focuses on RCTs of GERD treated by traditional Chinese herbal formula combined with western medicine versus western medicine only, and the type of western medicine, dosage, and the duration of treatment will not be limited.

#### 2.3.4 Type of outcome measures

The primary outcome is improvement in the symptom scores, and the secondary outcomes included the symptom scores after treatment, the response rate associated with the treatment, and the rate of relapse and adverse reactions.

### 2.4 Exclusion criteria

(1) Studies with unsatisfactory outcome indicators, or the outcomes of interest were not clearly reported;
(2) Studies with the most complete data will be selected for duplicate published studies;
(3) Extracting the related data from the published results is impossible, and unable to obtain the primary data after contacting the author;
(4) Literature with errors in random methods.

### 2.5 Search strategy

The literature will be systematically searched using the PubMed, Embase, Medline, Cochrane Library, China National Knowledge Infrastructure (CNKI), Wanfang database, Chongqing VIP Chinese Science and Technology Periodical Database, and Chinese Biological and Medical database (CMB) until Aug. 28, 2020. The reference lists of RCTs will be reviewed for eligible inclusion. The search terms and combination of keywords are “Chinese herbal formula,” “traditional Chinese medicine,” “gastroesophageal reflux disease,” “acid reflux,” etc, which will also be revised according to the rules of different database. The search strategy of PubMed is listed in Table 1.

**Table 1:**
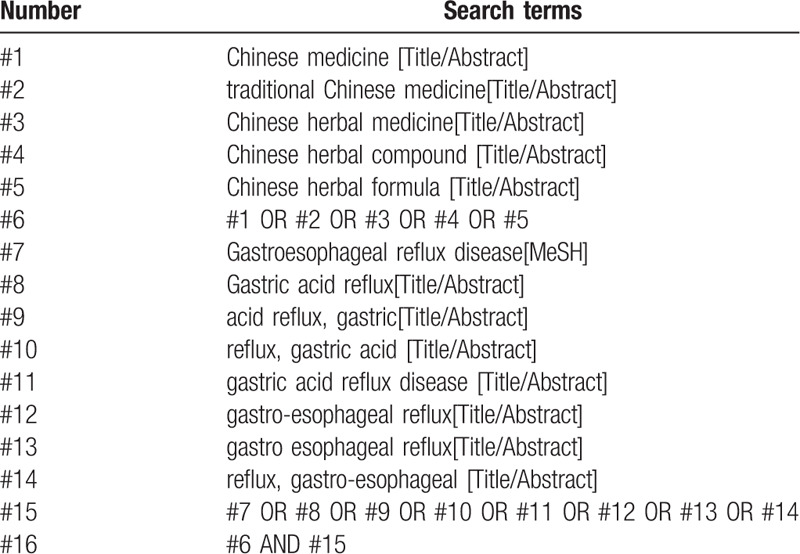
Search strategy in PubMed database.

### 2.6 Data extraction

**Figure 1:**
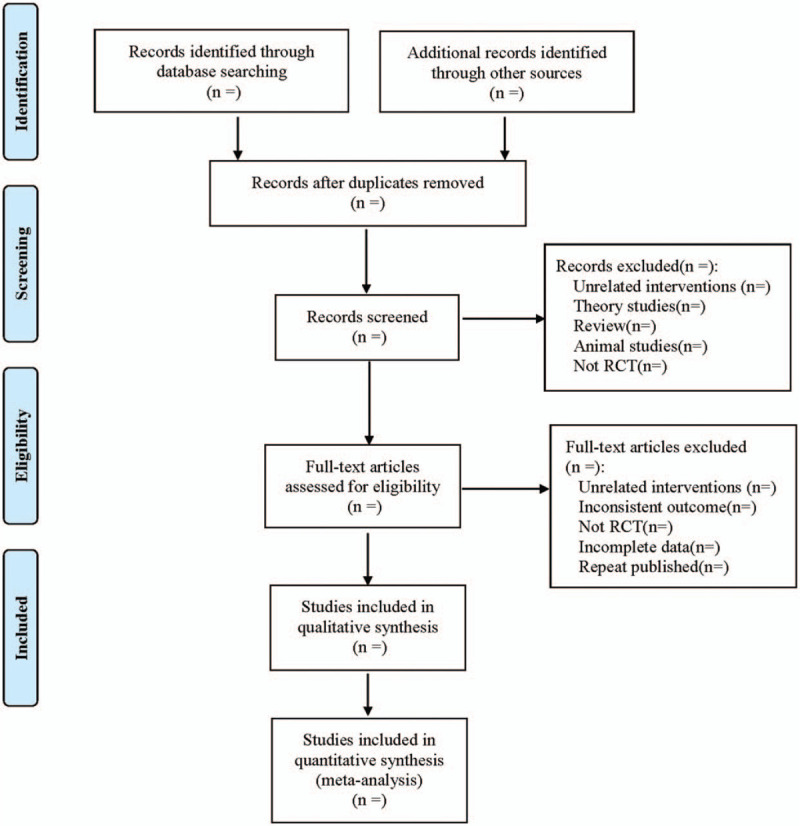
Flow diagram.

The literature will be managed by Endnote X7. The literature screening process is shown in Figure 1. Two reviewers will separately complete the extraction. Under the instruction of the Cochrane Collaboration, the following data from the eligible RCTs will be extracted into Excel 2019: study identification (title, authors, journal, publication year and country), participants information (age, sample size, sex ratio, course of disease), randomization method, concealment, interventions in treatment and control groups, outcomes, adverse events, and other details. When discrepancies appear, a third reviewer will be consulted.

### 2.7 Risk of bias assessment

The risk of bias in RCTs will be evaluated independently by 2 researchers in accordance with the Cochrane Handbook of Systematic Reviewers, including: random sequence generation, allocation concealment, blinding of participants and personnel, blinding of outcome assessment, incomplete outcome data, selective reporting, and other bias. The quality of studies is classified as being at of high, unclear or low risk of bias. In case of disagreement, a third researcher will be consulted to make the decision.

### 2.8 Statistical analysis

#### 2.8.1 Data synthesis

Statistical analysis will be conducted with the RevMan 5.3 software provided by the Cochrane Collaboration.

1. Relative risk is selected as the statistic for the dichotomous variable. Weighted mean difference is estimated for continuous variables when the tools and units of measurement indicators are the same, and standardized mean difference is estimated with different tools or units of measurement, and all the above are represented by effect value with a 95% Confidence interval.
2. Heterogeneity test: *Q* test is used to qualitatively determine inter-study heterogeneity. A *P* value ≥.1 is considered as no inter-study heterogeneity, and a *P* value <.1 is considered as inter-study heterogeneity. At the same time, *I*^*2*^ value is used to quantitatively evaluate the inter-study heterogeneity. An *I*^*2*^ score ≤50%, is considered to be a good heterogeneity, and the fixed-effect model is adopted. An *I*^*2*^ value >50% is considered to be significant heterogeneity, and the source of heterogeneity will be explored through subgroup analysis or sensitivity analysis. If there is no obvious clinical or methodological heterogeneity, it will be considered as statistical heterogeneity, and the random-effect model will be used for analysis. Descriptive analysis will be used if there is significant clinical heterogeneity between the 2 groups and subgroup analysis is not available.

#### 2.8.2 Dealing with missing data

If data is missing or incomplete in a study, the corresponding author will be contacted to obtain the missing data. If impossible, the study will be removed.

#### 2.8.3 Heterogeneity and subgroup analysis

In order to reduce the clinical heterogeneity between studies, subgroup analysis is conducted according to the nonerosive GERD and erosive esophagitis GERD.

#### 2.8.4 Sensitivity analysis

In order to test the stability of meta-analysis results of indicators, a one-by-one elimination method will be adopted for sensitivity analysis.

#### 2.8.5 Reporting bias

For the major outcome indicators, funnel plot will be used to qualitatively detect publication bias when the number of included study is ≥10. Egger and Begg test are used to quantitatively assess potential publication bias.

#### 2.8.6 Evidence quality evaluation

The Grading of Recommendations Assessment, Development, and Evaluation (GRADE) will be used to assess the quality of evidence, as high, moderate, low, and very low.

## 3 Discussion

Gastroesophageal reflux disease (GERD) is often encountered in clinical practice, and it is considered as one of the most common gastrointestinal diseases. Proton-pump inhibitors, such as omeprazole, are thought to be the most effective western medications, followed by H2 receptor blockers, such as ranitidine,^[12]^ which could improve the quality of life of patients with GERD, but with many adverse events. In China, Traditional Chinese herbal formula has history-proven benefits for GERD,^[13]^ and clinical studies have proved the efficacy and safety of Chinese herbal formula in treating GERD.^[9,10]^ Mechanism research found that Chinese herbal formula could relieve dilated intercellular spaces and desmosome disruption in esophageal epithelium, protected mitochondria from fragmentation to significantly resist esophageal morphology changes in ovalbumin-induced and acid exposure rat model.^[14]^ Also, they could improve the pH value of gastric contents, decrease the gastrointestinal hormones, and improve the inflammatory damage in rats.^[15]^ A combination of Chinese herbal formula and western medicine against GERD could significantly improve the treatment effect and reduce the side effects of western medicine.

This will be the first systematic review and meta-analysis to comprehensively compare the efficacy and safety of traditional Chinese herbal formula combined with western medicine versus western medicine for GERD. This meta-analysis and systematic review will help to determine potential benefits of Chinese herbal formula combined with western medicine compared with different western medicines to against GERD. However, there are some inevitable limitations. Our search did not include studied in other languages except Chinese and English, which might result in certain selective bias. In addition, the potentially high heterogeneity among different clinical studies might also influence the final results.

## Author contributions

**Data curation:** Lin Wuhong, Xirong Liu, Huasheng Lin.

**Funding support:** Guihua Huang.

**Investigation:** Guihua Huang.

**Literature retrieval:** Heng Zhou and Chunbing Feng.

**Resources:** Heng Zhou.

**Software:** Chunbing Feng, Tingshuai Wang, Renjiu Liang.

**Supervision:** Guihua Huang.

**Writing – original draft:** Wuhong Lin.

**Writing – review & editing:** Wuhong Lin, Guihua Huang.

## Abbreviations

Abbreviations: GERD = gastroesophageal reflux disease, RCTs = Randomized controlled trials.

## References

[1] Kethman W Hawn M. New approaches to gastroesophageal reflux disease. J Gastrointest Surg 2017;21:1544–52. 28623447 10.1007/s11605-017-3439-5

[2] Chen J Brady P. Gastroesophageal reflux disease: pathophysiology, diagnosis, and treatment. Gastroenterol Nurs 2019;42:20–8. 30688703 10.1097/SGA.0000000000000359

[3] Singendonk M Goudswaard E Langendam M. Prevalence of gastroesophageal reflux disease symptoms in infants and children: a systematic review. J Pediatr Gastroenterol Nutr 2019;68:811–7. 31124988 10.1097/MPG.0000000000002280

[4] Wang K Zhang L He ZH. A population-based survey of gastroesophageal reflux disease in a region with high prevalence of esophageal cancer in China. Chin Med J 2019;132:1516–23. 31045906 10.1097/CM9.0000000000000275 PMC6616241

[5] Akinola MA Oyedele TA Akande KO. Gastroesophageal reflux disease: prevalence and Extraesophageal manifestations among undergraduate students in South West Nigeria. BMC Gastroenterol 2020;20:160. 32456613 10.1186/s12876-020-01292-1 PMC7251857

[6] Maret-Ouda J Wahlin K Artama M. Risk of esophageal adenocarcinoma after antireflux surgery in patients with gastroesophageal reflux disease in the Nordic countries. JAMA Oncol 2018;4:1576–82. 30422249 10.1001/jamaoncol.2018.3054 PMC6248086

[7] Holmberg D Ness-Jensen E Mattsson F. Endoscopy for gastroesophageal reflux disease and survival in esophageal adenocarcinoma. Int J Cancer 2020;147:93–9. 31583704 10.1002/ijc.32721

[8] Dai YK Wu YB Wen H. Different traditional herbal medicines for the treatment of gastroesophageal reflux disease in adults. Front Pharmacol 2020;11:884. 32765255 10.3389/fphar.2020.00884 PMC7378538

[9] Shih YS Tsai CH Li TC. Effect of wu chu yu tang on gastroesophageal reflux disease: Randomized, double-blind, placebo-controlled trial. Phytomedicine 2019;56:118–25. 30668332 10.1016/j.phymed.2018.09.185

[10] Li S Huang M Wu G. Efficacy of Chinese herbal formula Sini Zuojin Decoction in treating gastroesophageal reflux disease: clinical evidence and potential mechanisms. Front Pharmacol 2020;11:76. 32174826 10.3389/fphar.2020.00076 PMC7057234

[11] Shamseer L Moher D Clarke M. Preferred reporting items for systematic review and meta-analysis protocols (PRISMA-P) 2015: elaboration and explanation. BMJ (Clinical research ed) 2015;350:g7647. 10.1136/bmj.g7647 25555855

[12] Kahrilas PJ Shaheen NJ Vaezi MF. American Gastroenterological Association Institute technical review on the management of gastroesophageal reflux disease. Gastroenterology 2008;135:1392–413. 413.e1-5. 18801365 10.1053/j.gastro.2008.08.044

[13] Ling W Li Y Jiang W. Common mechanism of pathogenesis in gastrointestinal diseases implied by consistent efficacy of single Chinese medicine formula: a PRISMA-compliant systematic review and meta-analysis. Medicine 2015;94:e1111. 26166106 10.1097/MD.0000000000001111 PMC4504579

[14] Jia B Xie C Wang Z. The effect of Heweijiangni-decoction on esophageal morphology in a rat model of OVA-induced visceral hypersensitivity followed by acid exposure. Cell Mol Biol (Noisy-le-Grand, France) 2019;65:73–8. 31304910

[15] Qiu Y Hu J Zhao C. Zhujie Hewei granules ameliorated reflux esophagitis in rats. Evid Based Complement Alternat Med 2019;2019:1392020. 31949463 10.1155/2019/1392020 PMC6944957

